# Insights into the Lignocellulose-Degrading Enzyme System of *Humicola grisea* var. *thermoidea* Based on Genome and Transcriptome Analysis

**DOI:** 10.1128/Spectrum.01088-21

**Published:** 2021-09-15

**Authors:** Andrei Stecca Steindorff, Luana Assis Serra, Eduardo Fernandes Formighieri, Fabrícia Paula de Faria, Marcio José Poças-Fonseca, João Ricardo Moreira de Almeida

**Affiliations:** a Laboratory of Genetics and Biotechnology, EMBRAPA Agroenergy, Brasília, Brazil; b Department of Biochemistry and Molecular Biology, Federal University of Goiás, Goiânia, Goiás, Brazil; c Department of Cellular Biology, University of Brasília, Brasília, Brazil; d Graduate Program of Microbial Biology, Department of Cell Biology, Institute of Biology, University of Brasília, Brasília, Brazil; University of Minnesota

**Keywords:** *Humicola grisea*, genome sequencing, transcriptome, sugarcane bagasse, pH regulation, CAZy enzymes

## Abstract

Humicola grisea var. *thermoidea* is a thermophilic ascomycete and important enzyme producer that has an efficient enzymatic system with a broad spectrum of thermostable carbohydrate-active (CAZy) enzymes. These enzymes can be employed in lignocellulose biomass deconstruction and other industrial applications. In this work, the genome of H. grisea var. *thermoidea* was sequenced. The acquired sequence reads were assembled into a total length of 28.75 Mbp. Genome features correlate with what was expected for thermophilic Sordariomycetes. The transcriptomic data showed that sugarcane bagasse significantly upregulated genes related to primary metabolism and polysaccharide deconstruction, especially hydrolases, at both pH 5 and pH 8. However, a number of exclusive and shared genes between the pH values were found, especially at pH 8. *H. grisea* expresses an average of 211 CAZy enzymes (CAZymes), which are capable of acting in different substrates. The top upregulated genes at both pH values represent CAZyme-encoding genes from different classes, including acetylxylan esterase, endo-1,4-β-mannosidase, exoglucanase, and endoglucanase genes. For the first time, the arsenal that the thermophilic fungus *H. grisea* var. *thermoidea* possesses to degrade the lignocellulosic biomass is shown. Carbon source and pH are of pivotal importance in regulating gene expression in this organism, and alkaline pH is a key regulatory factor for sugarcane bagasse hydrolysis. This work paves the way for the genetic manipulation and robust biotechnological applications of this fungus.

**IMPORTANCE** Most studies regarding the use of fungi as enzyme producers for biomass deconstruction have focused on mesophile species, whereas the potential of thermophiles has been evaluated less. This study revealed, through genome and transcriptome analyses, the genetic repertoire of the biotechnological relevant thermophile fungus *Humicola grisea*. Comparative genomics helped us to further understand the biology and biotechnological potential of *H. grisea*. The results demonstrate that this fungus possesses an arsenal of carbohydrate-active (CAZy) enzymes to degrade the lignocellulosic biomass. Indeed, it expresses more than 200 genes encoding CAZy enzymes when cultivated in sugarcane bagasse. Carbon source and pH are key factors for regulating the gene expression in this organism. This work shows, for the first time, the great potential of *H. grisea* as an enzyme producer and a gene donor for biotechnological applications and provides the base for the genetic manipulation and robust biotechnological applications of this fungus.

## INTRODUCTION

The microbial production of a variety of fuels and chemicals from the lignocellulose biomass sugars has been evaluated extensively as an alternative to fossil fuels (1–3). In this conversion process, the fermentable sugars need to be released from cellulose and hemicellulose present in the biomass cell wall by pretreatment and hydrolysis (4). In the enzymatic hydrolysis, lignocellulolytic enzymes from filamentous fungi have received great attention, as these microorganisms are highly efficient in biomass hydrolysis for the production capacity of both specific enzymes and enzymatic cocktails (5).

A few industrially relevant fungi species have been well characterized in terms of genetics and physiology, especially mesophilic species, such as *Trichoderma* spp., *Penicillium* spp., *Aspergillus* spp., *Neurospora* spp., *Phanerochaete* spp., and *Trametes* spp. (6). A great extent of thermophilic filamentous fungi (more than 50 species) is capable of producing enzymes that act synergistically for the degradation of lignocellulosic biomass; however, relatively few studies aiming to characterize and unveil their enzymatic potential have been reported so far (6). Enzymes from thermophilic fungi, like Humicola grisea, Thielavia terrestris, Myceliophthora thermophila, and Malbranchea cinnamomea, are of interest because they tend to be more thermostable than enzymes from mesophilic fungi (7–10).

Several carbohydrate-active (CAZy) enzymes, i.e., enzymes that can degrade, modify, or create glycosidic bonds (11), from thermophilic microorganisms have been identified, purified, and characterized in recent years. Genomic studies have allowed the prospection of a diversity of enzymes used to deconstruct the plant cell wall in T. terrestris, M. thermophila, and M. cinnamomea (12, 13). Furthermore, transcriptome and secretome analyses demonstrated the differential regulation and secretion of CAZymes produced by the different species, which will vary according to the substrate employed (14, 15).

In general, biomass deconstruction requires the expression of different classes of putative CAZymes. The expression of glycoside hydrolases, including cellulases, hemicellulases, pectinases, and others, is broadly regulated by the carbon source (14). For instance, *M. thermophila* is capable of secreting 95 glycoside hydrolases (GH), but the production of each protein depends on the carbon source employed in the cultivation (15). Also, the CAZymes expression can be influenced by the medium pH that is closely regulated by the PacC transcription factor. Several studies have demonstrated that PacC modulates lignocellulolytic enzyme production in species such as Aspergillus nidulans, Trichoderma reesei, *and* H. grisea var. *thermoidea* (16, 17).

The ascomycete *Humicola grisea var. thermoidea* was isolated from Brazilian soil (18), and it belongs to the Sordariomycetes class and the Sordariales order. The genus *Humicola* was described in 1914 by Traaen for the species Humicola fuscoatra and *H. grisea*. The *Humicola* species are capable of growing in a diverse set of substrates, such as soil, decomposing plant biomass, and agriculture residues (19). Recently, the genus *Humicola* was revised through a thorough phylogenetic analysis and *H. grisea* was renamed Trichocladium griseum. This fungus is considered thermophilic because it can grow in moderate to high temperatures, with optimal growth of around 40 to 42°C (20).

Several studies reported that *H. grisea* var. *thermoidea* produces a wide range of thermostable CAZymes, such as cellulases (21), glucoamylase (22), beta-glucosidases (23), xylanases (24), feruloyl esterases (25), and chitinases (26). In this view, *H. grisea* var. *thermoidea* represents a promising microorganism for application in different industrial processes, such as plant biomass deconstruction (27); recycled paper (28), detergents (29), and food processing (25). Additionally, there are extensive reports about the potential of *H. grisea var. thermoidea* as an efficient hydrolase gene donor that can be cloned and expressed in diverse heterologous hosts (7, 30, 31).

To unveil the global hydrolytic potential of *H. grisea* var. *thermoidea*, its genome was sequenced and annotated for the first time in this work. Transcriptome analysis was also performed after *H. grisea* growth on an inducing (sugarcane bagasse) and a repressing (glucose) carbon source at pH 5 or pH 8. The results allowed the determination of the genetic repertoire of this fungus and the comparison with other fungi from the Sordariomycetes class.

## RESULTS

### *Humicola grisea* genome features.

**FIG 1:**
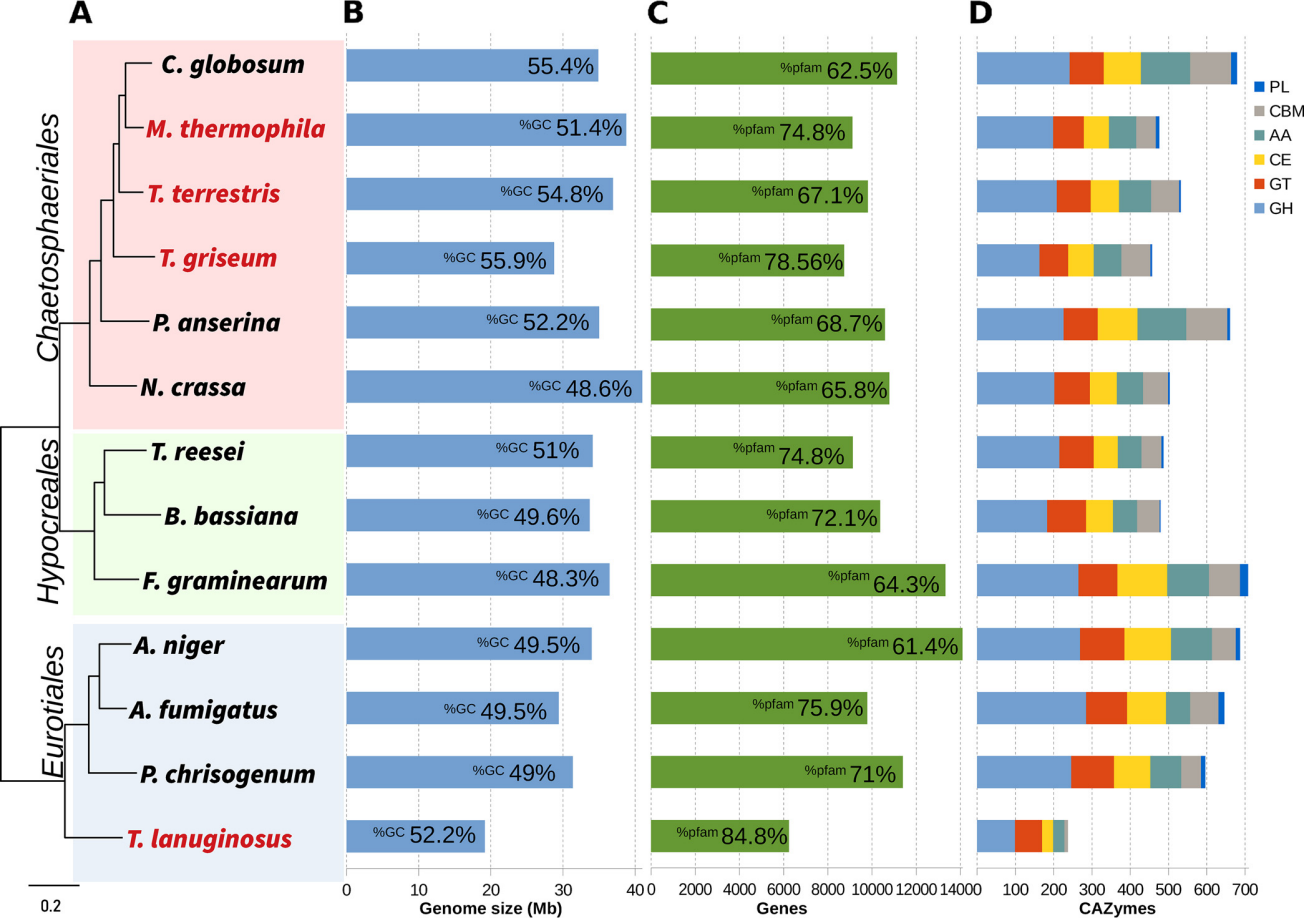
Genome features of 13 Ascomycetes genomes. (A) The RAxML maximum-likelihood phylogenetic tree using 200 single-copy genes shows the three orders Sordariales, Hypocreales, and Eurotiales. All nodes have maximum support value. Thermophile genomes are shown in red. (B) Genome size and percentage of GC of each genome. (C) Gene count and percentage of genes with the presence of at least one Pfam domain. (D) Distribution of CAZyme families among the genomes. CAZymes: auxiliary activities (AA), carbohydrate-binding module (CBM), carbohydrate esterases (CE), glycoside hydrolases (GH), glycosyl transferases (GT), and polysaccharide lyases (PL).

The genome of *H. grisea* var. *thermoidea* was sequenced in an Illumina-based whole-genome shotgun sequencing approach. This resulted in 9,460,608 paired reads of 2 by 150 bp, with an approximate insert size of 350 bp combined with 6,837,917 mate-paired reads of about 3,000 bp long. The acquired sequence reads were assembled into 33 scaffolds with a total length of 28.75 Mb (Table 1). This size is smaller than that described for the thermophilic neighbors *M. thermophila* (38.74 Mb) and *T. terrestris* (36.91 Mb) and the mesophilic Chaetomium globosum (34.9 Mb) and Neurospora crassa (39.9 Mb) (Table 1; Fig. 1A). Despite the small size, the genome seems close to its finishing size, showing 98.2% of completeness (accessed with BUSCO v2.0.1 analysis).

**TABLE 1:** Genome features of *H. grisea* var. *thermoidea* and genome comparison with other filamentous fungi

Feature	*H. grisea* var. *thermoidea*	*T. terrestris*	*C. globosum*	N. crassa	*T. reesei*	A. nidulans	*T. lanuginosus*
Genome size	28.75 Mb	36.91 Mb	34.9 Mb	39.9 Mb	34.1 Mb	31.67 Mb	23.3 Mb
Scaffolds	33	6	37	21	89	91	6
*N*_50_	1.805 Mb	9.477 Mb	4.721 Mb	6.00 Mb		2.44 Mb	4.00 Mb
GC content	55.9%	54.8%	55.4%	48.6%	51.0%	50.3%	52.2%
Genes	8,736	9,813	11,232	10,812	9,143	9,541	5,105
Secreted proteins	781	789	862^*a*^	592^*a*^		704^*a*^	
Completeness	98.20%			97%	99%	95%	98%
References	This work	13	59	38	35	60	61

a Data from Lum and Min, 2011 (62).

Genome structural and functional annotation was performed using *ab initio* predictors and homology to proteins and transcripts from Sordariomyceta, as well as data from the RNA-seq experiment realized in this study (see Materials and Methods). Gene modeling yielded 8,736 coding sequences, a smaller number than that of other fungi from the Sordariales. Indeed, this number is relatively close to that of *M. thermophila*, 9,110, but around 20% lower than the number of predicted genes in N. crassa (10,620) and Chaetomium globosum (11,124) (Fig. 1C). The important cellulose-degrading enzyme producer T. reesei, Hypocreales, presents a slightly superior number of predicted genes (Fig. 1C).

The protein domains encoded by *H. grisea* genome were compared with those of other fungi using InterProScan and SignalP4.1. Like other Sordariales, *H. grisea* showed a Pfam domain in approximately 67% of the putative carried genes (Fig. 1D). A total of 781 proteins are potentially secreted, including CAZymes and proteases (Table 1). These comparisons must be made with caution due to the different approaches used to generate gene/protein models in different projects. The GC content is the highest among the related fungi (Fig. 1B). This could be related to thermophilism and the high gene density of the genome (303.9 genes/Mb) (13).

### *Humicola grisea* CAZyme genes.

To have a better insight into the *H. grisea* biomass degradation potential, the genes encoding CAZymes of fungi from Sordariales, Hypocreales, and Eurotiales were compared (Fig. 1D) (Supplemental File 1). In the CAZy database classification (32, 33), proteins are grouped based on their similarity in amino acid sequences, catalytic mechanisms, and structural characteristics. Those families are auxiliary activities (AA), carbohydrate-binding module (CBM), carbohydrate esterases (CE), glycoside hydrolases (GH), glycosyl transferases (GT), and polysaccharide lyases (PL) (34).

*H. grisea* possesses a vast number of genes encoding carbohydrate-active enzymes (a total of 435), comparable in number with those of *M. thermophila* and *T. reesei* (Fig. 1D). The fungi that primarily consume monosaccharides, like Saccharomyces cerevisiae, Kluyveromyces lactis, and Yarrowia lipolytica, have around 120 CAZymes (14). Among cell wall degraders, this number varies considerably. For instance, compared to the other members of Sordariales, *H. grisea* has only 159 genes encoding GH. In contrast, *T. reesei* is capable of producing 200 GHs, whereas N. crassa (produces 171 GHs), Aspergillus niger, and Aspergillus fumigatus (Eurotiales) are among the bigger producers, reaching almost 300 GHs (Fig. 1D) (35).

The importance of complex carbohydrates as nutrients for *H. grisea* is demonstrated by the number of GH (159), CE (66), AA (71), CBM (54), and PL (4) (Fig. 1D), as well as the number of genes specifically related to degradation of plant-based polysaccharides (Table 2), found in its genome. In general, these numbers are close to those of other members of the Sordariales family, like N. crassa, *M. thermophila*, and *T. terrestris*, but slightly lower than those of *C. globosum*, a fungus that showed the higher number of CAZymes in this order (Fig. 1D; Table 2). However, significant differences among the enzyme families can be found among the fungi. While *T. reesei* showed a higher number of GH and GT than did *H. grisea*, it possessed fewer AA (61), CBM (45), and CE (61) enzymes. These observations corroborate with previous reports of the relatively lower number of hemicellulases produced by *T. reesei*, which does not produce tannase and feruloyl esterase (35). On the other hand, *H. grisea* is an efficient hemicellulose degrader (7, 25, 36, 37).

**TABLE 2:** Number of genes related to degradation of plant-based polysaccharides^*a*^

Genome	Cellulose	Xylan	Galactomanan	Xyloglucan	Pectin	Starch	Inulin
*T. lanuginosus*	4	8	1	4	5	12	1
Penicillum chrisogenum	12	9	11	5	28	28	6
A. fumigatus	18	15	11	8	50	24	5
A. niger	14	10	9	8	48	19	5
Fusarium graminearum	21	29	6	9	39	18	6
Beauvaria bassiana	3	16	3	5	3	12	1
*T. reesei*	9	10	11	7	6	10	0
N. crassa	24	15	3	3	15	15	2
Podospora anserina	49	54	5	4	12	14	0
*H. grisea*	39	36	2	4	7	12	0
*T. terrestris*	33	28	3	5	20	14	1
*M. thermophila*	34	31	4	4	16	14	0
*C. globosum*	59	47	5	7	22	14	1

a Gene numbers related to degradation of different plant-based polysaccharides detected in the selected genomes according to De Vries et al., 2017 (63). Cellulose: GH5_4, GH5_5, GH5_22, GH6, GH7, GH45; xylan: GH10, GH11, GH62, GH67, GH115, CE1, CE15; galactomannan: GH5_7, GH26, GH27, GH36, GH134; xyloglucan: GH12, GH29, GH74, GH95; pectin: GH28, GH53, GH78, GH88, GH93, GH105, PL1, PL3, PL4, PL9, PL11, PL22, CE8, CE12; starch: GH13_1, GH13_5, GH13_40, GH15, GH31, GH133; inulin: GH32.

### Differential gene expression during *H. grisea* growth on sugar cane bagasse in different pH values.

The genome sequencing and annotation of *H. grisea* demonstrated the fungus’s genetic repertoire of cell wall-degrading enzymes. To identify the genes involved in *H. grisea* early growth in lignocellulosic biomass, a genome-wide RNA-seq transcriptional profiling was used. Cultivations were carried out using milled sugarcane bagasse as an inducing carbon source and glucose as a repressing one, at both pH 5 and pH 8. A total of 323,849,916 sequence reads were obtained after quality trimming and then aligned onto the reference genome. The principal-component analysis (PCA) of samples and replicates based on expression patterns using the DESeq2 package reveals the discrimination between samples (three biological replicates for each condition) and the good quality and reproducibility of the data (Supplemental File 2).

**FIG 2:**
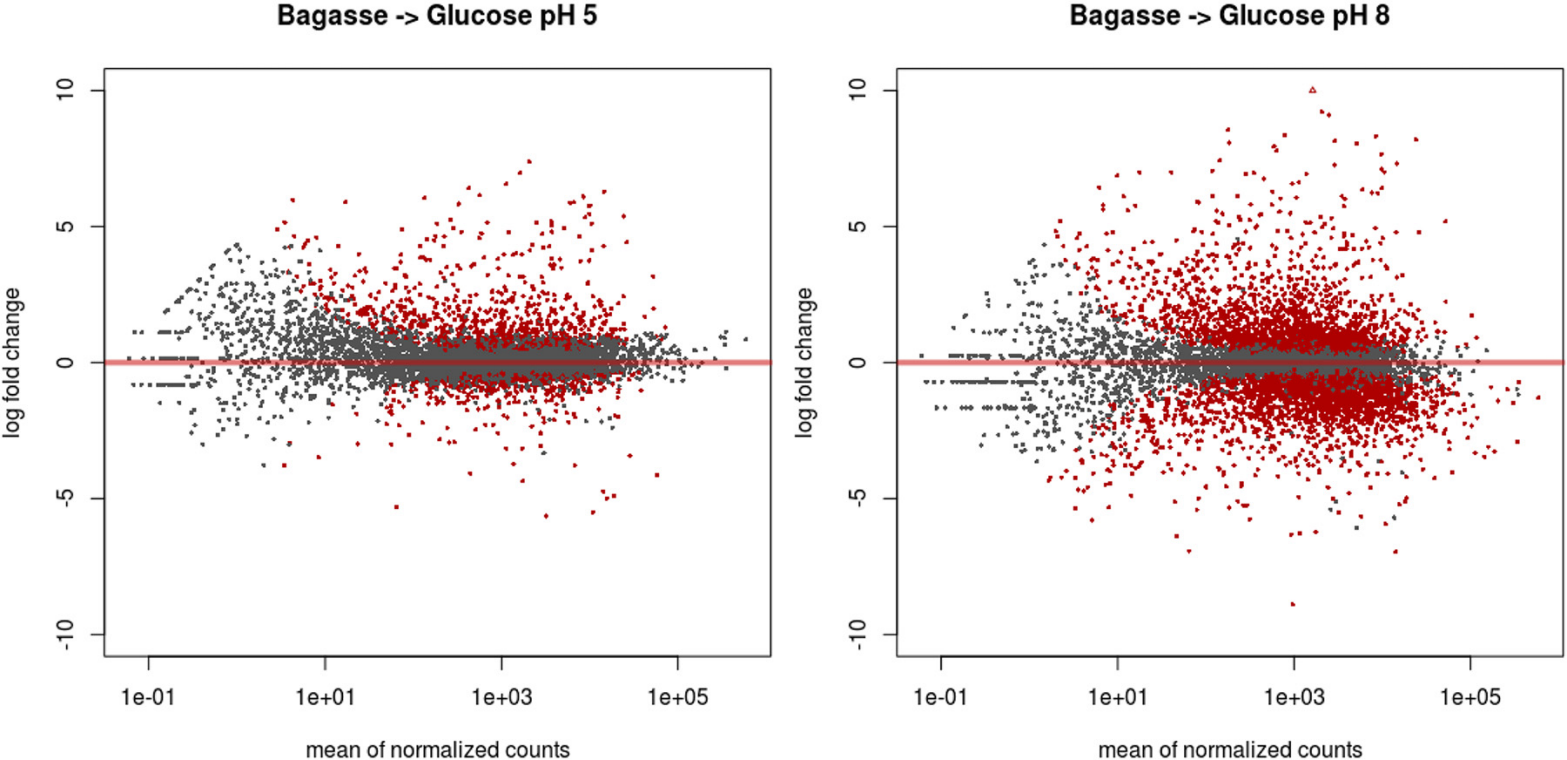
Distribution of gene expression of *H. grisea* grown in sugarcane bagasse normalized with growth on glucose as the sole carbon source. The two pH values, pH 5 (A) and pH 8 (B), exhibited differentially expressed genes (*P* value adjusted of <0.05) showed in red when normalized with glucose.

**FIG 3:**
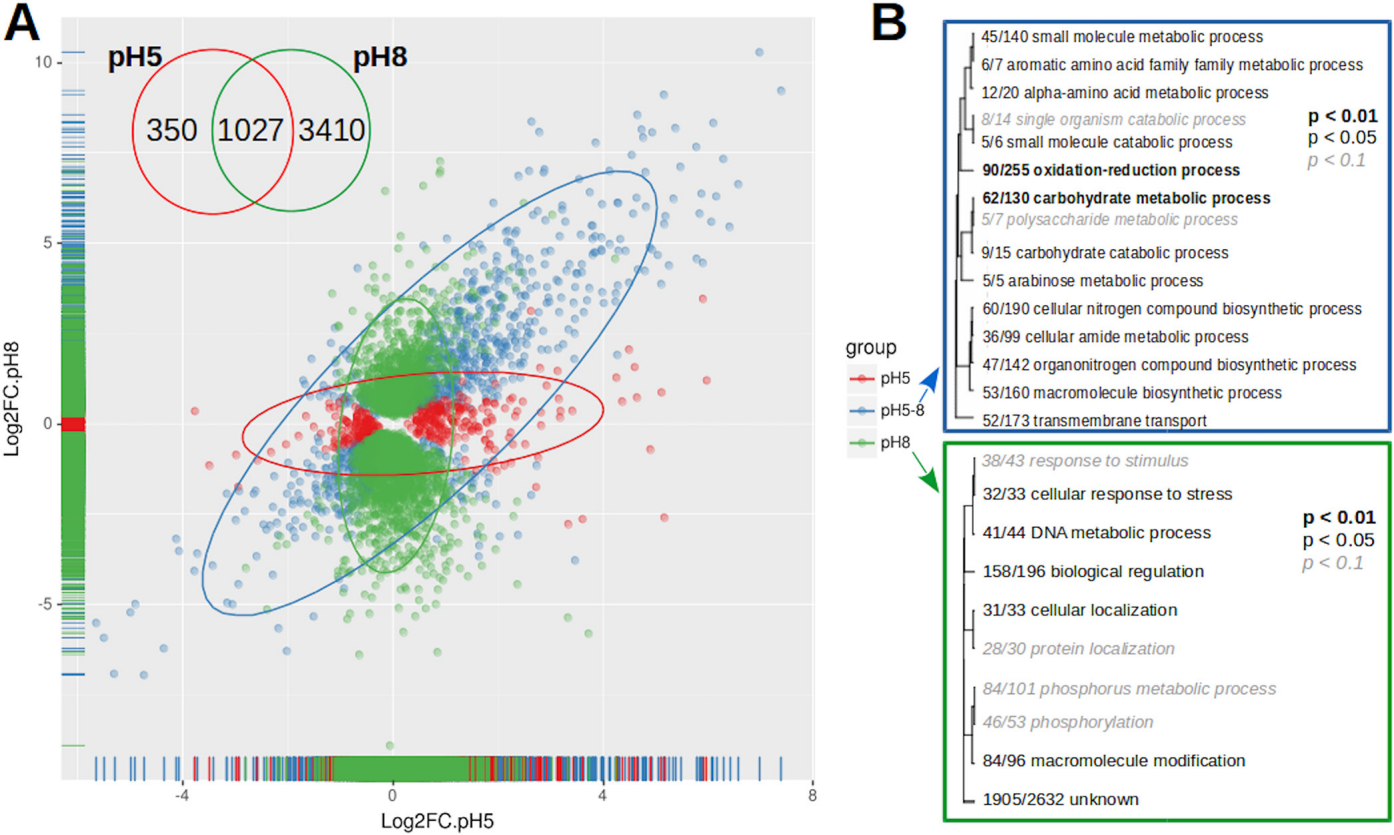
Differentially expressed genes at pH 5, pH 8, or both values. (A) Scatterplot of differentially expressed genes at pH 5 or pH 8. The colors denote genes expressed at pH 5 (red), pH 8 (green), and both conditions (blue). The Venn diagram shows the number of exclusive and shared genes between the pH values. (B) Fisher enrichment analysis of GO terms on each condition compared to the whole genome as background. No category exclusively enriched at pH 5 was found.

To map the differentially expressed genes, the data on sugarcane were normalized with the data using glucose as the sole carbon source. The MAplots in Fig. 2 show the distribution of *H. grisea* transcripts at pH 5 and pH 8. Growth at pH 8 resulted in a number of differentially expressed genes higher than that of growth at pH 5 (4,438 and 1,376, respectively). At pH 5, 838 genes were upregulated and 539 were downregulated. On the other hand, at pH 8, 2,032 genes were upregulated and 2,405 were downregulated. Figure 3A shows that 350 genes were exclusively differentially expressed at pH 5, whereas 3,410 genes were exclusively expressed at pH 8. A total of 1,027 genes were differentially expressed at both pH values. These genes show the highest fold change in expression, and some of them are related to carbohydrate metabolic and catabolic processes (Fig. 3).

Functional categories were assigned to the differentially expressed genes according to Gene Ontology (GO). To enrich the category analysis for up- and downregulated genes at each pH, a Fisher exact test (*P* < 0.05) was performed (Fig. 3B). Categories related to the oxidation-reduction process and carbohydrate metabolic process and others related to the primary metabolism were significantly upregulated at both pH 5 and pH 8. Any category was exclusively upregulated at pH 5, whereas categories’ cellular response to stress and DNA metabolic process were upregulated at pH 8 (Fig. 3B). On the other hand, downregulated categories showed a larger diversity of functions: RNA metabolism, transmembrane transporter, and electron carrier.

The top 10 upregulated genes at both pH values represent genes encoding CAZymes: cellulases, xylanases, mannanases, AA9 enzymes, and esterases (Table 3). Furthermore, the expression of these transcripts was further increased at pH 8. For instance, for the endo-β-1,4-glucanase (EGLD) gene, the log_2_FC for pH 8 was 9.11 compared with the log_2_FC of 5.16 at pH 5. Among these classes of enzymes, the expression of acetylxylan esterase-, endo-1,4-β-mannosidase-, exoglucanase-, and endoglucanase-encoding genes can be observed at both pH values. Indeed, most of the transcripts in Table 3 correspond to CAZy GH and AA families.

**TABLE 3:** Expression of top 10 genes differentially expressed and upregulated at pH 5 and pH 8

Putative genes (blast best-hit)	log_2_FC pH 5	log_2_FC pH 8	CAZy annotation
Top 10 genes upregulated at pH 5			
*axe1* acetylxylan esterase	7.39	9.22	CE5-CBM1
*manA* endo-1,4-beta-mannosidase	6.97	10.27	GH26-CBM35
cel1 cellulose-growth-specific protein	6.57	6.63	AA9
*bxlB* exo-1,4-beta-xylosidase	6.40	5.46	GH3
*eglD* endo-beta-1,4-glucanase D	6.29	7.32	CBM1-AA9
Pectin lyase-like protein	6.15	6.28	
*gux1* exoglucanase 1	6.10	8.32	GH7
*pme* pectinesterase	6.06	5.58	CE8
Hypothetical protein	5.97	1.19	
FSH1 serine hydrolase	5.90	3.46	
Top 10 genes upregulated at pH 8			
*manA* endo-1,4-beta-mannosidase	6.97	10.27	GH26-CBM35
*axe1* acetylxylan esterase	7.39	9.22	CE5-CBM1
*eglD* endo-beta-1,4-glucanase D	5.16	9.11	AA9
CUTI cutinase	4.65	8.55	CE5
Hypothetical protein	2.64	8.35	AA9
*gux1* exoglucanase 1	6.10	8.32	GH7
LIP3 secreted lipase	5.37	8.19	CE10
*xyn2* endo-1,4-beta-xylanase 2	4.73	8.14	GH11
*ganA* arabinogalactan endo-beta-1,4-galactanase	2.78	8.08	GH53
*manC* mannan endo-1,4-beta-mannosidase C	4.93	8.05	GH5

### CAZy enzymes expression.

**FIG 4:**
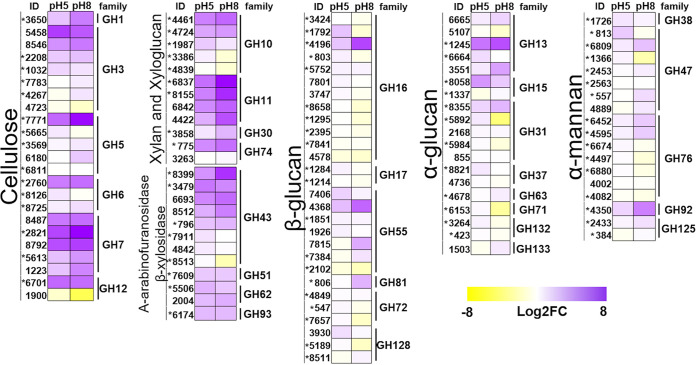
Expression of putative genes encoding glycoside hydrolases (GH) separated by the likely substrate. The asterisk (*) means the gene is potentially regulated by the PacC regulator.

To better understand the transcriptional regulation of genes encoding cell wall-degrading enzymes, we evaluated the differential expression of the CAZy family’s genes during growth in sugarcane bagasse presence. Fig. 4 shows the expression of glycoside hydrolases according to the predicted enzyme-substrate, whereas Supplemental File 3 shows the expression data set for all CAZy families. For cellulose, six of GH’s families were expressed by *H. grisea* (1, 3, 5, 6, 7, and 12). However, the genes in these families exhibited different expression patterns (up- and downregulated at different pHs) (Fig. 4). Families GH3 and GH7 are the biggest ones, represented by seven and five enzymes, respectively. Family GH7 encompasses key enzymes for biomass degradation, such as endo-β-1,4-glucanases, endo-β-1,3-glucanases, and reducing end cellobiohydrolases. The GH3 family comprises enzymes like β-glucosidase, xylan 1,4-β-xylosidase, and α-l-arabinofuranosidase. Only two transcripts from the GH12 family, including an endoglucanase, were shown as differentially expressed (Fig. 4).

For the degradation of β-glucan, most of the enzyme-encoding genes did not show differential expression. Only few genes from the GH16 and GH55 families were upregulated at pH 8 (Fig. 4). Specifically, the genes 4196 and 4368, which encode glucan endo-1,3-beta-glucosidase A1 and glucan 1,3-beta-glucosidase, showed the highest fold change in expression, 6.11 and 5.04, respectively, for the families associated with degradation of β-glucan (Fig. 4; Supplemental File 3). A similar pattern of nonsignificant differential expression was verified for genes related to α-glucan and α-mannan degradation (Fig. 4). For α-glucan as the substrate, one transcript for the GH13 family (ID1245), encoding an alpha-amylase A, was upregulated at both pH values, whereas ID 3551, encoding an alpha-glucosidase, was upregulated only at pH 8 (Fig. 4). Families GH31 and GH71 each presented one gene downregulated at pH 8. For α-mannan as the substrate, one sequence (ID 1366), corresponding to the GH47 family (α-mannosidase), was also downregulated at pH 8. The GH92 family, composed of different types of mannosyl and mannosidases, presented the upregulation of one transcript (ID 4350) at pH 8.

For the xylan and xyloglucan substrates, four GH families (10, 11, 30, and 74) presented down- or upregulated transcripts upon *H. grisea* growth on sugarcane bagasse (Fig. 4). Five genes from the GH10 family, comprising endoxylanases and xylan endotransglycosylases, were differentially regulated. The GH11 family genes (endo-β-1,3-xylanase and endo-β-1,4-xylanase) were upregulated. For arabinofuranosidase and β-xylosidase as the substrates, genes encoding four enzyme families were expressed, with GH43 (β-xylosidase and α-l-arabinofuranosidase) demonstrating the four most expressed transcripts but also the less expressed one (ID 8513) (Fig. 4). As with other substrates, some genes from the same family presented different expression patterns. These results corroborate previous reports that demonstrate the *H. grisea* efficiency in hemicellulose degradation (7, 24, 25, 36). Comparatively, the genome annotations of *T. reesei* and N. crassa indicate that they can produce 16 and 19 hemicelluloses (GH families 10, 11, 26, 29, 43, 51, 53, 54, 62, 67, 74, and 95), compared with the 24 of *H. grisea* (35, 38).

Additionally, to evaluate the pH-dependence expression of lignocellulolytic enzymes in *H. grisea* var. *thermoidea* more broadly, the expression profiles of CAZymes in pH 5 and pH 8 were compared, and the genes potentially under the control of the transcription factor pacC were identified by searching the *pacC*-binding consensus 5′-GCCARG-3′ within the upstream region in each corresponding gene. From the 387 genes putatively encoding CAZymes identified in the transcriptome of *H. grisea*, 191 present a domain to *pacC* (Supplemental File 3) (Fig. 4). The potential broad regulation of PacC on putative glycoside hydrolase-encoding genes in *H. grisea* var. *thermoidea* is shown in Fig. 4, which is in good agreement with the increased number of genes upregulated at pH 8 (Fig. 3). The pH signaling cascade in A. nidulans has at least six members (*palA*, *palB*, *palC*, *palF*, *palH*, and *palI*), which are also present in the *H. grisea* genome (17). However, the expression pattern of this signaling cascade is not clear in the transcriptome data, with only *palI* being differentially expressed in bagasse normalized with glucose at pH 8. The *palF* gene is found in the genome, but no transcriptome reads were mapped to this region.

## DISCUSSION

This study brings for the first time insights into the genome of the fungus *H. grisea* var. *thermoidea* and reveals its enzymatic potential for the degradation of plant biomass through the analysis of its transcriptome. Regarding the genome, the size is smaller than that described for the thermophilic neighbors, and a high GC content is similar to that of other fungi within the family. The reduction of the genome size is a characteristic strongly associated with thermostability in fungi, as well as a high GC content (13, 39). The genomic and transcriptomic analysis demonstrated that *H. grisea* var. *thermoidea* represents a promising microorganism for application in plant biomass deconstruction. It possesses a wide range of putative CAZymes, several of which are related to plant-based polysaccharides degradation. Indeed, several thermostable enzymes of *H. grisea* targeting biomass deconstruction (mainly cellulose and hemicellulose) have been expressed and characterized (7, 21–26, 30, 31).

Among the 211 putative genes encoding CAZy enzymes identified in the transcriptome analysis of *H. grisea*, the most expressed transcripts were cellulases (endoglucanases and cellobiohydrolases) and hemicellulases (especially xylanases), which correlate well with the enzyme activities required for growth on sugarcane bagasse as the sole carbon source. When the secretome of *T. reesei* and A. niger was analyzed upon growth on sugarcane biomass, the GH families involved in the deconstruction of celluloses 3, 5, 6, 7, and 12 were found in A. niger, whereas GH 3, 5, 6, and 7 were secreted by *T. reesei* (40). In comparison, all of these GH’s families were expressed by *H. grisea*. A higher variety of enzymes is necessary for the degradation of the hemicellulose because it contains different types of sugar chains, such as arabinoxylan, β-glucan, and xyloglucan. In the secretome of A. niger and *T. reesei*, the families GH10 and GH11 (endoxylanases), GH3 (β-xylosidase), GH43, GH51, and GH54 (arabinofuranosidases), and GH35 (galactosidases) were found (40, 41). In comparison, in *H. grisea*, GH10, 11, 43, and 51 were also expressed. However, these comparisons should be made with caution because the CAZy response can be different because of the experimental conditions (i.e., different compositions of sugarcane biomass), and the expression data may not correlate with the number of enzymes secreted.

In addition, the transcriptome analysis of *H. grisea* revealed a consistent upregulation of AA9 proteins when the fungus was cultivated in sugarcane bagasse in both pH 5 and pH 8. This could suggest a synergism between AA9 and GHs families expressed differentially since these proteins enhance the activity from one another. The transcriptome of the *T. terrestris* LPH172 showed abundantly expressed AA9 lytic polysaccharide monooxygenase (LPMO) genes in Avicel, rice straw, and beechwood xylan. The presence of LPMO-encoding genes in thermophilic fungus confirms the importance of (AA9) LPMOs for plant biomass decomposition (39). Currently, several studies have demonstrated the action of oxidative enzymes, such as lytic polysaccharide monooxygenases (LPMOs) classified as AA, capable of degrading cellulose together with cellulases (42–44). Moreover, a recent study demonstrated the boosting effect of recombinant hemicellulases (endoxylanase-HXYN2 and β-xylosidase-HXYLA) from *H. grisea* together with an α-l-arabinofuranosidase (AFB3) from Penicillium purpurogenum in the hydrolysis of sugarcane bagasse, exhibiting the potential of these enzymes from *H. grisea* to compose enzymatic consortiums for biomass hydrolysis (45).

The expression of several glycoside hydrolases (Table 3; Fig. 4) of *H. grisea* var. *thermoidea* was further increased at pH 8. These results corroborate the refined time course expression profile established for *H. grisea* var. *thermoidea* glycoside hydrolase-encoding genes when the fungus was grown at different pH values and distinct carbon sources performed by Mello-de-Sousa and collaborators (46). These authors described an early parallel increase in mRNA accumulation for *cbh*1.1, *cbh*1.2, *egl*1, *egl*2, *egl*3 (endoglucanase), bgl4 (beta-glucosidase), and *xyn*1 (xylanase) genes at alkaline milieu (pH 8.0) with sugarcane bagasse as the sole carbon source. A distinct profile was observed for the endoglucanase *egl*4 transcripts, which preferably accumulated in acidic conditions (46). In addition, electrophoretic mobility shift assays (EMSAs) indicated that the CreA and PacC transcription factors are involved in the carbon source and pH regulation, respectively, of *H. grisea* var. *thermoidea* cellulase genes (46). Similar to what happens in *H. grisea*, a variable number of genes encoding cellulases and hemicellulases are affected not only by available carbon source but also by the pH of the culture medium due to regulation at the transcriptional level of PacC in *T. reesei*, A. fumigatus, A. nidulans, and N. crassa (17, 47, 48). These findings of genome and transcriptome information enable comparative studies to better understand the molecular mechanisms, the metabolic changes, and the evolution of different species within this group of fungi.

### Conclusions.

This is the first description of important aspects of the biology, physiology, and evolution of the thermophilic fungus *H. grisea* var. *thermoidea* using genome sequencing and genome-wide transcriptome analysis. The 28.75 Mb genome contains 8,736 putative genes and is smaller than others from Sordariomycetes. The GC content is similar to that of the other species within the Chaetomiaceae family, suggesting a correlation with thermophilism. The transcriptome analysis revealed that alkaline pH is a key regulatory factor for glycoside hydrolases. The expression of 211 different genes for CAZy enzymes when cultivated in sugarcane bagasse demonstrates the great arsenal that *H. grisea* possesses to degrade the lignocellulosic biomass. This work paves the way for the genetic manipulation and robust biotechnological applications of this fungus.

## MATERIALS AND METHODS

### Fungal strain.

The fungus *Humicola grisea* var. *thermoidea* isolated from Brazilian soil (18) was maintained at 42°C on 4.0% (wt/vol) oatmeal (Quaker) solid medium. For mycelium obtainment, 10^6^ spores/ml were inoculated in 50 ml of Pontecorvo’s minimal medium (MM) (49), at pH 6.8 (nonbuffered), enriched with 0.25% (wt/vol) yeast extract and 0.1% (wt/vol) peptone and supplemented with 1% (wt/vol) glucose. Incubation proceeded for 24 h (42°C/120 rpm).

### Cultivation.

The conditions employed were similar to those described in Mello-de-Souza et al. (46). Briefly, *H. grisea* var. *thermoidea* was cultivated at 42°C on 4.0% (wt/vol) oatmeal (Quaker) solid medium without photoperiod. For mycelial growth, 10^6^ conidia/ml were inoculated in 50 ml of Pontecorvo’s minimal medium (MM), enriched with 0.25% (wt/vol) yeast extract and 0.1% (wt/vol) bacterial peptone, and supplemented with 1% (wt/vol) glucose at pH 6.8 (nonbuffered). The incubation occurred at 42°C, 120 rpm, for 24 h. The mycelium produced was used for DNA extraction and to initiate transcriptome experiments.

For the transcriptome experiment, pregrown mycelium from 12 flasks was filtered, washed with sterile water, and transferred to fresh 50 ml MM, supplemented with 1% (wt/vol) glucose (GLU) or 0.1% (wt/vol) ball-milled, steam-exploded sugarcane bagasse (SCB) as the sole carbon sources. The culture medium pH was adjusted to 5.0 or 8.0 (buffered with 100 mM sodium citrate). Based on a previous report (46) that demonstrated the early induction of cellulases and xylanases of *H. grisea* grown in SCB, cultures were incubated for 6 h at 42°C, 120 rpm. Then, mycelia were harvested, washed with cold sterile water, drained, frozen in liquid nitrogen, and stocked at −80°C. In total, 12 independent samples were collected, three biological samples for each culture condition (GLU pH 5, GLU pH 8, SCB pH 5, and SCB pH 8).

### DNA and RNA isolation.

The mycelia obtained from the cultivation in MM were immediately ground in liquid nitrogen into a fine powder. DNA was isolated using the DNAzol reagent (Invitrogen), according to the manufacturer’s instructions. For RNA extraction, the mycelia from the 12 samples (3 biological replicates) were grounded in liquid nitrogen into a fine powder and RNA was isolated using the TRIzol reagent (Invitrogen) following the manufacturer’s instructions.

RNAs were quantified by spectrophotometry and the integrity was evaluated by electrophoresis in 1.0% agarose gel stained with 0.5 μg ml^−1^ ethidium bromide. The RNA samples were then treated with DNase I (RQ1 RNase-free DNase-Promega).

### Genome assembly and annotation.

*H. grisea* var. *thermoidea* genomic DNA (gDNA) was sequenced by two strategies: short inserts (Illumina Hiseq2000 paired-end 2 by 150 bp) and mate pairs (Illumina Hiseq2000 paired-end 2 by 100 bp with an average insert size of 3,000 bp). FastQC (https://www.bioinformatics.babraham.ac.uk/projects/fastqc/) was used to evaluate the libraries quality before and after trimming. For quality trimming and sequence filtering, the tophat NGS QC Toolkit was employed to remove sequencing adapters’ residues and low-quality reads.

The assembly was performed with AllPaths-LG (https://software.broadinstitute.org/allpaths-lg/blog/) using a maximum coverage of 100× for each library. Genome structural and functional annotation was performed with the MAKER pipeline (50) using three *ab initio* predictors: Augustus (51), SNAP (52), and GeneMark-ES (53). Two data sets of proteins and transcripts from Sordariomycetes retrieved from the RefSeq/GenBank were used as structural support, as well as Trinity-assembled transcripts derived from the RNA-seq experiment described in “Transcriptome analysis.” Functional annotation of the predicted genes was made using InterProScan v.5.21.60 with embedded PFAM v29, Gene Ontology, InterProScan, and SignalP4.1 programs/databases.

CAZymes were predicted based on the dbCAN v6.0 HMMs pipeline. Transporters were predicted based on Transporter Classification Database – TCDB (http://www.tcdb.org/) and transcription factors based on DBD - Transcription factor prediction database (54), both using minimum criteria of an E value of <1e^−10^ and identity of >35% on blastp analysis. Genome completeness was accessed with BUSCO v2.0.1 using the Sordariomycetes core data set (55). Complete genome assembly and annotation were deposited at DDBJ/EMBL/GenBank under accession QQBE00000000.

### Analysis of protein family evolution.

The evolution of CAZymes family size variation (expansion or contraction) was analyzed by CAFE (56) using as input an ortholog table generated by OrthoFinder (57) and CAZy annotation, with a *P* value of 0.01 and applying a stochastic model of gene death and birth.

### Transcriptome analysis.

Illumina Hiseq2000 100 bp paired-end reads were used for transcript quantification. Quality-filtered reads were mapped to the *H. grisea* assembled in this work using the TopHat2 v2.0.4 aligner (http://ccb.jhu.edu/software/tophat), and HTSeq version 0.6.0 was used to count reads mapped to *H. grisea* genome. The R package DESeq2 version 1.6.3 was used to perform the differential expression analysis, using the raw number of reads mapped to each gene in each sample to perform statistical tests, based on the negative binomial distribution, which indicates whether a gene is differentially expressed in a condition relative to another gene. Therefore, the DESeq2 package was utilized for normalization, using the median log deviation, and for the differential expression analysis, applying an adjusted *P* value of ≤0.05 as the threshold. Functional enrichment analysis of differentially expressed genes based on Gene Ontology (GO) terms was performed using the R package GO_MWU (https://github.com/z0on/GO_MWU). The RNA data set was deposited at DDBJ/EMBL/GenBank under accession PRJNA717364.

### Genes under *pacC* regulation.

In order to detect genes potentially under the transcription factor *pacC* control, we generated a FASTA file with 1,500 bp upstream (5′ UTR) from each gene and then the detection of *pacC*-binding consensus 5′-GCCARG-3′ within the region (58). A one-sided enrichment test (Fisher exact test) was performed, and after false-discovery rate correction, none of the samples were significantly enriched in bagasse and not pH.

### Data analysis.

The genome and transcriptome data sets generated and analyzed during the current study are available in the GenBank (https://www.ncbi.nlm.nih.gov/) under accession numbers QQBE00000000 and PRJNA717364.
