# Supplementary material for: Insights into the Lignocellulose-Degrading Enzyme System of Humicola grisea var. thermoidea Based on Genome and Transcriptome Analysis
Source: Microbiol Spectr. 2021 Sep 15;9(2):e01088-21. doi: 10.1128/Spectrum.01088-21 (PMC8557918; doi:10.1128/Spectrum.01088-21)
Supplement: SUPPLEMENTAL FILE 3 — Supplemental material. Download SPECTRUM01088-21_Supp_2_seq2.pdf, PDF file, 0.04 MB [file spectrum01088-21_supp_2_seq2.pdf]

Supplemental Material 2

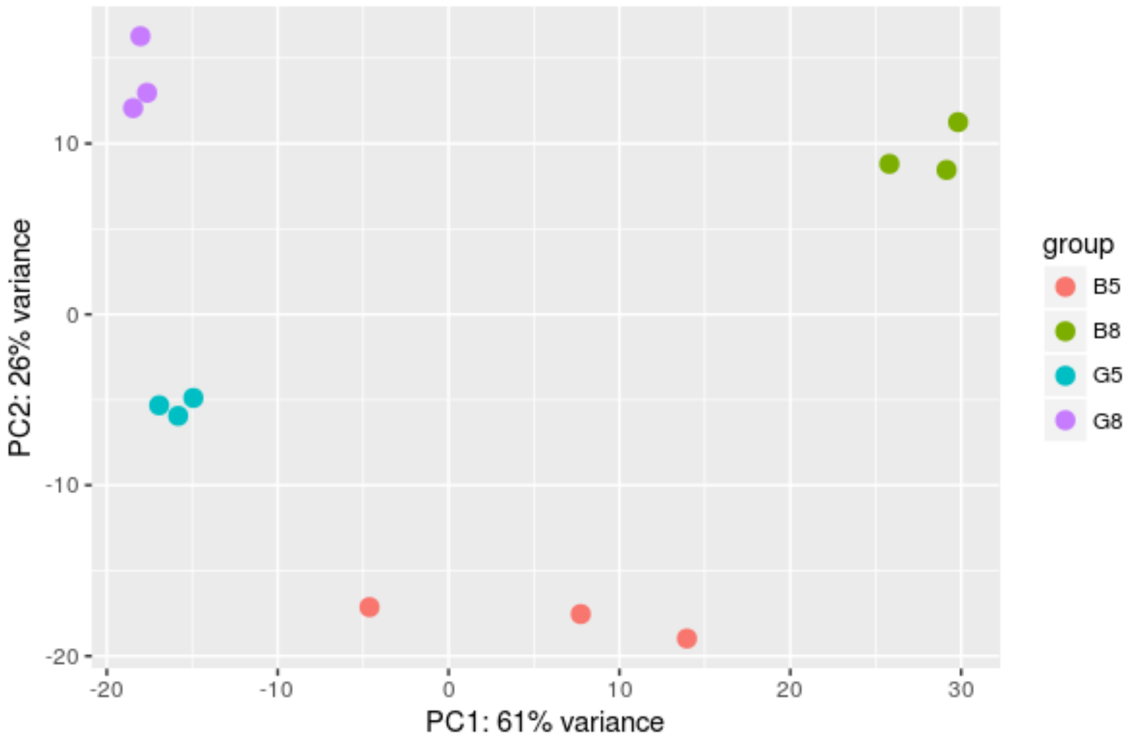

Supplementary Figure 1. **Principal component analysis (PCA) of genome-wide transcriptional response of *H. grisea* grown in sugarcane bagasse and glucose at pH 5 or pH 8.** The log-transformed counts from biological replicates. B5: sugarcane bagasse pH 5, B8: sugarcane bagasse pH 8, G5: glucose pH 5 and G8: glucose pH 8.
